# 1148. Long-term Persistence of Measles and Rubella Virus Neutralizing Antibody Levels among Adults with Three Doses of Measles-Mumps-Rubella Vaccine

**DOI:** 10.1093/ofid/ofad500.989

**Published:** 2023-11-27

**Authors:** Oluwakemi Alonge, Mona Marin, Carole J Hickman, Sun B Sowers, Min-hsin Chen, Lijuan Hao, Joseph Icenogle, David L McClure, Stephen N Crooke, Huong McLean

**Affiliations:** Marshfield Clinic Research Institute, Marshfield, Wisconsin; Centers for Disease Control and Prevention, Atlanta, Georgia; CDC, Grayson, Georgia; Centers for Disease Control and Prevention, Atlanta, Georgia; Centers for Disease Control and Prevention, Atlanta, Georgia; Viral Vaccine Preventable Diseases Branch, Atlanta, Georgia; Centers for Disease Control and Prevention, Atlanta, Georgia; Marshfield Clinic Research Institute, Marshfield, Wisconsin; Centers for Disease Control and Prevention, Atlanta, Georgia; Marshfield Clinic Research Institute, Marshfield, Wisconsin

## Abstract

**Background:**

A third dose of measles-mumps-rubella vaccine (MMR) may be administered for various reasons, but there are limited data on long-term immunogenicity. We assessed persistence of measles and rubella neutralizing antibodies among adults after receipt of three MMR doses.

**Methods:**

Adults who received two MMR doses in childhood and a third dose as young adults (aged 18-28 years) in 2009-2010 were recalled at around 5 (2014-2016) and 9-11 (2019-2021) years after receipt of the third MMR dose. Measles and rubella antibody levels were assessed by plaque-reduction and soluble immunocolorimetric neutralization assays, respectively. Participants with measles antibody concentrations < 120 mIU/ml or rubella antibody concentrations < 10 U/mL were considered potentially susceptible to infection. Generalized estimating equations models were used to estimate geometric mean concentrations (GMC) and 95% confidence intervals (CI) of antibody levels over time.

**Results:**

Approximately 5 and 9-11 years after receipt of the third MMR dose, 408 (aged 22-33 years) and 304 (aged 26-37 years) adults were reassessed, respectively. For measles, GMC was 428 mIU/mL (95% CI, 392 – 468 mIU/mL) at 5 years after the third MMR dose, declining to 381 mIU/mL (95% CI, 339 – 428 mIU/mL) at 11 years after the third MMR dose (Fig 1). Measles antibody concentrations during both long-term follow-up periods declined to levels lower than before receipt of the third dose (Fig 2). At the last follow-up visit (2019-2021), 10% of participants were potentially susceptible to measles infection compared to 3% before receipt of the third MMR dose up to 11 years earlier. For rubella, GMCs were stable throughout the long-term follow-up period (63 U/mL to 65 U/mL) (Fig 3). Rubella antibody concentrations during the long-term follow-up periods remained higher than levels before receipt of the third MMR dose and were similar to levels at 1 year after receipt of the third MMR dose (Fig 2). None of the participants were susceptible to rubella at the last follow-up visit.

**Figure:**
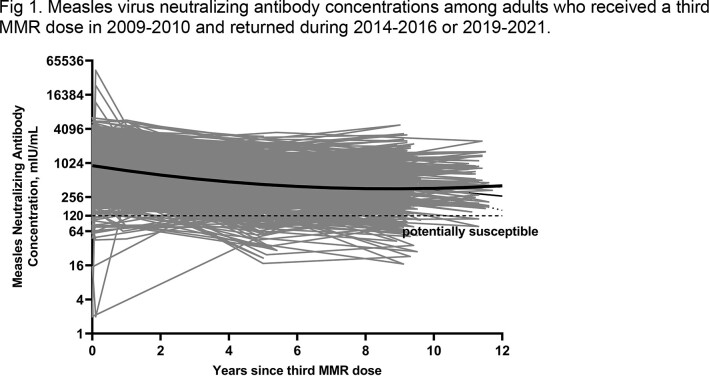

The solid black line shows the trend in population GMC estimates from before vaccination (0 year) through 12 years after receipt of the third MMR dose. The gray lines show the measles virus antibody concentrations over time for each individual. The dotted horizontal line shows concentration <120 mIU/mL used as the cutoff for susceptibility.

**Figure:**
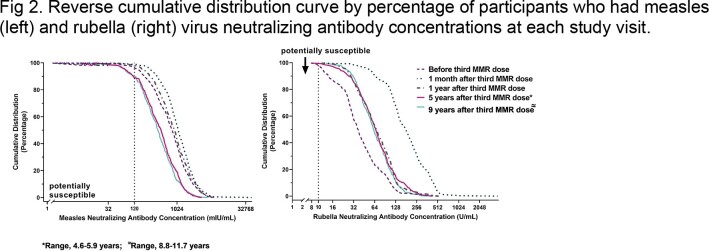

The vertical dotted lines represent the cutoff for susceptibility.

**Figure:**
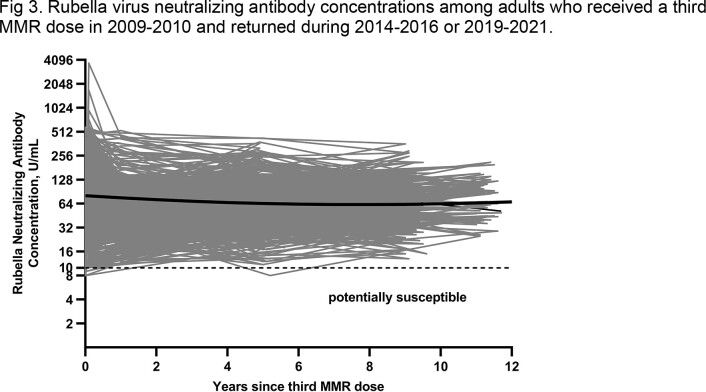

The geometric mean concentrations (GMCs) were estimated using generalized estimating equations model. The solid black line shows the trend in population GMC estimates from before vaccination (0 year) through 12 years after receipt of the third MMR dose. The gray lines show the rubella virus antibody concentrations over time for each individual. The dotted horizontal line shows concentration <10 U/mL used as the cutoff for susceptibility.

**Conclusion:**

Neutralizing antibody levels to measles and rubella remained high and persisted through 11 years after vaccination among adults who received three MMR doses. However, some adults may become susceptible to measles infection over time despite receipt of three MMR doses.

**Disclosures:**

**Stephen N. Crooke, PhD**, Bionano Genomics: Stocks/Bonds|Caribou Biosciences: Stocks/Bonds|Pfizer: Stocks/Bonds|Vistagen Therapeutics: Stocks/Bonds **Huong McLean, PhD, MPH**, Seqirus: Grant/Research Support

